# Is Assessing the Presence of NASH by Liver Histology or Surrogate Markers Always Advisable?

**DOI:** 10.5812/hepatmon.7560

**Published:** 2013-02-20

**Authors:** Giovanni Tarantino

**Affiliations:** 1 Department of Clinical and Experimental Medicine, School of Naples, Federico II University Medical, Naples, Italy

**Keywords:** Liver, Histology, Biological Markers

To gather objective evidence for the effectiveness of their interventions, should responsible clinicians rely on the necessity to better characterize patients with nonalcoholic fatty liver disease as being affected by the benign or progressive form (simple fatty liver or nonalcoholic steatohepatitis) and by which means? Disease markers, imaging tools, histology? Just here, the Author discusses the weaknesses and the strengths of this approach in every-day practice. Insulin resistance (IR) are due to hepatic lipotoxicity, which is consequence of the increased flux of free fatty acids towards the liver. Non-alcoholic fatty liver disease (NAFLD) is a primary hepatic disease that should be treated in its more severe entity, i.e., nonalcoholic steatohepatitis (NASH) and for this reason it is essential to distinguish this latter form from the benign one, i.e., simple fatty liver (FL). The gold standard in order to obtain this diagnosis is represented by liver biopsy. Alternatively, when the invasive procedure is risky, the likely correct diagnosis is reached by imaging tools/biochemical markers. NAFLD is present also in non-obese patients. It was that lifestyle of hunting and gathering that shaped us; constant and daily activity was stamped into our genes, as was a dependence on fresh foods (the only kinds that were available). Our human ancestors during the Palaeolithic period had a diet based on roots, nuts, vegetables, fruit, meat, organ meats (liver, spleen, heart, tongue, kidneys, brains and intestines) and insects. A diet in less concordance with our evolutionary legacy (agrarian diet coupled to an increased salt consumption during modern times) has led to high prevalence of both obesity/type 2 diabetes (T2D) and non-alcoholic fatty liver disease (NAFLD) among Western ethnic groups. Due to insufficient adaptation, a possible mechanism might be the potential to alter glucose homeostasis (insulin resistance, IR). To confirm this hypothesis, afret adjusting for age, baseline BMI and lifestyle factors such as exercise and sleep duration, Mozaffarian et al found that the foods most associated with weight gain over a four-year period were French fries, potato chips, sugary drinks, meats, sweets and refined grains (1). This point raises more questions than answers: Is obesity the main cause of IR, worsened by ectopic fat storage in liver, or is a hepatic “primitive” metabolic defect to determine IR? Encompassing NAFLD a broad spectrum of illnesses ranges from FL to NASH, cryptogenic liver cirrhosis (likely post-NASH) and finally NAFLD-related hepato cellular carcinoma, histopathology merits have so far outstripped the clinical-laboratory ones in nosographically characterizing these entities. To date, histological assessment of liver steatosis, ballooning, lobular inflammation in H&E staining coupled to fibrosis detection is considered the best tool for diagnosis of NASH. As immediate observation, being liver fibrosis a dynamic process constituted by neo-fibrogenesis and collagenolytic activity, as a major mechanism of fibrosis resolution (2), its frozen evaluation casts some doubts. Secondly, is FL really benign? Evaluating the disease progression of NAFLD in a prospective study with paired liver biopsies at 3 years, patients with simple steatosis could still develop NASH and fibrosis progression. Weight reduction was associated with non-progressive disease (3). It is noteworthy to stress that hepatic steatosis could be per se pro-fibrogenic (4, 5). Is liver inflammation a strictu sensu injury to counteract with every effort or is it a response to the excessive caloric intake? Are programmed cellular death and mild necrosis really effects to blame or are they the starting points for liver regeneration? Changing the subject, "pure" alcoholic fatty liver has been widely assumed to be benign with very low risk of progression to cirrhosis. Nine out of 88 patients with a histological diagnosis of alcoholic fatty liver and no evidence of fibrosis or alcoholic hepatitis, followed for a median of 10.5 years, developed cirrhosis and a further seven patients showed fibrosis. Independent histological predictors of progression on index biopsy were the presence of mixed macro/micro vesicular fat and of giant mitochondria. Authors concluded that patients with these features should be counseled intensively regarding their alcohol consumption (6). We can no longer regard alcoholic fatty liver as benign, in the presence of continuing high alcohol consumption. Why do we suppose that it is unlikely (the progression) for patients with nonalcoholic simple fatty liver who continue to be obese or suffer from T2D, in absence of long-lasting epidemiological studies? Similarly, what is the basic mechanism that links the metabolic syndrome-associated co-morbidities, i.e., drug induced liver injury, NAFLD, obstructive sleep apnea syndrome, polycystic ovarian syndrome and alcoholic liver diseases? Hypoxia induced by mitochondrial dysfunction. To gather objective evidence for the effectiveness of their interventions, when dealing with NAFLD patients, should responsible clinicians rely on the necessity to better characterize them as being affected by the benign or progressive form and by which means? Despite a low rate of overall complications, percutaneous liver biopsy remains an invasive procedure with the risk of potentially lethal hemorrhage and infections and is further complicated in the growing number of obese patients with NAFLD. “Doctor, do I need to undergo liver biopsy, or don’t I?” These words could be heard through the walls of any hepatologist’s office quite often. Liver biopsy should be recommended only when the results would affect the treatment or the management of hepatic diseases due to the health risks. To overcome this limitation, clinical, instrumental and laboratory variables (readily/rarely available) help predict the presence of NASH (each researcher cheering on his/her proposed tool). Being the hepatocyte the ultimate adipocyte (7), intended as situs of ectopic fat storage, calories control is mandatory to reduce IR due to the low grade chronic inflammation, hallmark of obesity (8). Nobody pretends that achieving and maintaining an ideal weight is an easy thing to do. People struggle to keep weight off because they are surrounded by food, inundated with food messages and constantly presented with opportunities to eat. Human beings are the only species in the world that has figured out how to be in complete control of its own food supply. The challenge now is to make sure the food does not take control of us. The CDC recommends that all adults get a minimum of 150 minutes of moderate aerobic activity per week, or 75 minutes a week of a vigorous activity. Is it always compatible with our life style? What about drugs exclusively used on NASH patients? Patients on pioglitazone plus low hypo caloric diet (LHD) for six months showed a greater reduction in necroinflammation than patients in the placebo plus LHD group, but the reduction in fibrosis did not differ significantly (9). Again, serum ALT levels were reduced with vitamin E and with pioglitazone (along 96 weeks), as compared with placebo and both agents were associated with reductions in hepatic steatosis and lobular inflammation but not with improvement in the more important fibrosis scores (10). Neither vitamin E nor metformin at 72 and 96 weeks was superior to placebo in attaining the sustained reduction in ALT level (primary outcome) in patients aged 8-17 years. Compared with placebo, neither therapy demonstrated significant improvements on steatosis, inflammation, nor fibrosis as individual components at histology (secondary outcome, intentionally) (11). A recent meta-analysis showed that thiazolidinediones significantly improve ballooning degeneration, lobular inflammation, steatosis and combined necroinflammation, but not the key feature, i.e., fibrosis (12). All in all, prescribing to take some cups of coffee pro die (13) could turn out to be a good choice to counteract the risk of NAFLD. Being this the best piece of advice, should clinicians clutch at straws and suggest patients should undergo liver biopsy? On the contrary, they ought to focus on diagnosis of potential NAFLD-associated diseases and adequate evaluation of cardio-vascular risk. In medicine, “gold” standard test refers to a diagnostic test that is the best available under reasonable conditions. It does not have to be necessarily the best possible test (in case of NAFLD: autopsy or a two/three decade-follow-up) for the condition in absolute terms. The astonishing observation is that some studies have suggested a certain rate of disagreement or interpretational errors in evaluating the histology features. It is advisable to look at these figures. In a large cohort, on the basis of canonical criteria, definite NASH was diagnosed in 58.1%, borderline NASH in 19.5% and "not definite NASH” in 22%. The NAFLD activity score (NAS) was ≥ 5 in 50% and ≤ 4 in 49%; only 75% of biopsies with definite NASH had an NAS ≥ 5, whereas 28% of borderline and 7% of "not NASH" biopsies had NAS ≥ 5. Of biopsies with an NAS ≥ 5, 86% had definite NASH and 3% "not definite NASH". NAS ≤ 4 did not indicate benign histology; in fact, 29% had definite NASH and only 42% had "no definite NASH". This study was undertaken using a large dataset of prospectively obtained clinical data and results from liver biopsies blindly reviewed by a committee comprised of the pathologists from 9 different centers. The aim was to evaluate if the diagnosis of NASH made by the pathologists correlated with a threshold value of feature-based scores that comprise the NAS of ≥ 5. Authors do not recommend it beyond clinical trials (14). It is, however, increasingly apparent from ongoing and published studies that the numeric value of the composite NAS is considered by some investigators to be either “synonymous” with, or actually a replacement for, a microscopic diagnosis that is based on overall pattern of injury as well as the presence of additional lesions such as zonality of lesions, portal inflammation, and fibrosis. In fact, Hjelkrem et al claim that a NAS ≥ 4 has optimal sensitivity and specificity for predicting NASH, and is the recommended value for admission into an interventional trial for NASH (15). On the other hand, the diagnoses of NASH by the original NASH subtypes and by a current study's definition of NASH were in moderate agreement with NAS (κ = 0.47 and κ = 0.51, respectively) and only in fair to moderate with the Brunt’s criteria (κ = 0.365 and κ = 0.44, respectively) (16). Again, although the simple and reproducible NAS was found to be a useful pathologic grading system in Korean NAFLD patients, however, the proportion of borderline cases based on the NAS was high. The "wait and see" strategy is necessary for evaluating the long-term prognosis (17). Clinically important differences exist between community general pathologist and expert hepatopathologist in assessing NAFLD using the NAS scoring system. More studies are needed to investigate its suitability for community-based clinical practice (18). Some hepatologists could object to the attitude towards not differentiating NASH with and without fibrosis, and could make reasonable claims on the necessity of detecting fibrosis (presence/entity). Poynard et al state that, even if assessing liver fibrosis is traditionally performed by liver biopsy, it is an imperfect gold standard. Thus, non-invasive techniques, liver stiffness measurements and biomarkers are needed and their wide use is welcome (19). Anyway, there are two schools of thought and everyone shows grassroots studies in support of his/her own tendency. To lend credence to their results, some researchers in favor of histology try to couple more precise methods with the classic ones. In a current investigation on cases of NAFLD, Mori et al, in order to demonstrate that liver stiffness clearly correlates to fibrosis (stage F1-F4), performed histopathology but also and mainly morphometric analyses such as the Sirius red-positive fibrotic area and alpha-SMA-positive area (20). Nevertheless, there are some pitfalls also in imaging method. It is necessary to highlight that, even when liver stiffness measurement is feasible, high BMI values, which are a characteristic of NAFLD patients, negatively affect the diagnostic reliability (21). But, if we consider that BMI estimates and increased ALT levels (also discovered in simple FL) were independent factors associated with liver stiffness (22), we should infer that not all the features of fibrosis are necessarily linked to the NASH presence. On the other hand, it is surprising that a simple test, with no-adjunctive-cost, such as the FIB4 index (based on age, AST and ALT levels, and platelet counts) is so rarely adopted/validated, judging from literature data. In fact, it was superior to other tested noninvasive markers of fibrosis in Japanese patients with NAFLD, characterized by a high negative predictive value for excluding advanced fibrosis. Anyway, the Authors pinpoint that the small number of cases of advanced fibrosis in this cohort means that this study has limited power for validating the high cut-off point (23). It happens to us all physicians. We look out into the future, trying our best to make wise decisions, only to find ourselves staring into the teeth of ferocious and widespread uncertainties. Trying to find a "robust" strategy, given the increasing prevalence of NAFLD, noninvasive markers for NASH are proposed as a promising approach for screening reasons in every-day practice. To test their reliability, these surrogate markers are compared to liver biopsy as “gold” standard. According to the previously highlighted data on histology this procedure is far from being both accurate and precise. If our criterion of validity is not itself really valid are surrogate diagnosis techniques consequently reliable? Anyway, they represent a trilling discovery like the one (24) that will help us better figure out the mechanisms of NAFLD (in this case the inflammation). I hope we will never read the results of a study as the following. Investigating the validity of parental reports of a history of respiratory disease in their children a survey of the child’s General Practice records to see whether a diagnosis had been made was performed. Authors did not conclude that the questionnaire instrument was wrong, but that the records, the criterion, were inadequate (25). Finally, Aristotle claimed that the highest level of wisdom was phronesis (prudence). Phronesis is basically the ability to discern the correct action when there is insufficient scientific evidence to determine the absolute truth. This is clearly the case for histology (or surrogate tools/markers) and NASH in every-day practice. Waiting for a better understanding of mechanisms, clinicians could focus on curing the related co-morbitities of NAFLD and not on correctly diagnosing NASH.
